# Association between the cartilage area ratio derived from 3D MRI and patient-reported symptoms in medial knee osteoarthritis

**DOI:** 10.1186/s13018-026-06986-y

**Published:** 2026-06-01

**Authors:** Yusuke Aimono, Nobutake Ozeki, Hisako Katano, Takuya Takakuwa, Hideyuki Koga, Makoto Tomita, Ichiro Sekiya

**Affiliations:** 1 Center for Stem Cell and Regenerative Medicine, Department of Applied Regenerative Medicine, Institute of Science Tokyo, 1-5-45 Yushima, Bunkyo-ku, Tokyo, 113-8510 Japan; 2 Department of Joint Surgery and Sports Medicine, Graduate School of Medical and Dental Sciences, Institute of Science Tokyo, Tokyo, Japan; 3 School of Data Science, Graduate School of Data Science, Yokohama City University, Yokohama, Japan

**Keywords:** Knee osteoarthritis, Cartilage area ratio, Automated MRI analysis, Medial compartment, Numerical rating scale

## Abstract

**Background:**

Knee osteoarthritis (OA) is characterized by progressive cartilage deterioration in the medial compartment. Three-dimensional (3D) MRI enables quantitative assessment of cartilage morphology, and the cartilage area ratio is a sensitive metric that integrates focal defects. This study investigated associations between medial compartment cartilage area ratios and numerical rating scale (NRS) scores in patients undergoing high tibial osteotomy (HTO).

**Methods:**

We analyzed 176 patients who underwent HTO for medial knee OA. All had preoperative 3.0 T MRI and completed a 10-item NRS questionnaire. Total NRS was the sum of Q1–Q4 and Q6–Q10 (Q5 excluded; maximum 90). Cartilage area ratios for the medial femur and medial tibia were calculated using automated 3D MRI analysis, and the medial meniscus coverage ratio was also measured. Item-level associations were assessed using Spearman correlation with Holm correction. Multivariable linear regression of total NRS adjusted for age, sex, body mass index (BMI), and Kellgren–Lawrence (KL) grade was performed. Subgroup analysis in KL grade 3–4 patients and sensitivity analyses progressively excluding high cartilage area ratio values were conducted.

**Results:**

Total NRS scores showed significant negative correlations with both medial femoral (rs =  − 0.31) and medial tibial (rs =  − 0.26) cartilage area ratios. Item-level analyses showed the strongest correlations for instability during running (Q8) and grinding/noise (Q9). In multivariable regression, both medial femoral (standardized β =  − 0.245, *P* = 0.007) and medial tibial (standardized β =  − 0.226,  *P* = 0.012) cartilage area ratios remained independently associated with total NRS, together with BMI, while age, sex, and KL grade showed no significant associations. The KL grade 3–4 subgroup retained significant correlations (n = 95; femoral rs =  − 0.26; tibial rs =  − 0.26). Sensitivity analyses confirmed that the inverse correlations were not driven by ceiling effects. Meniscus coverage ratios correlated positively with cartilage area ratios (medial femoral rs = 0.41; medial tibial rs = 0.47) but not with total NRS.

**Conclusions:**

Lower cartilage area ratios in the medial compartment are independently associated with greater symptom severity, particularly instability during running and mechanical symptoms. This supports cartilage area ratios as clinically relevant structural biomarkers in knee OA.

## Background

Knee osteoarthritis (OA) is a leading worldwide cause of pain, functional limitation, and reduced quality of life. Structural deterioration of the articular cartilage, particularly in the medial compartment, plays a central role in disease progression and is a major determinant of symptoms and treatment strategies [1]. One established joint-preserving surgical option for medial knee OA is high tibial osteotomy (HTO), which aims to offload the medial compartment [2]. Although structural assessment is increasingly incorporated into clinical decision-making, the relationship between detailed cartilage morphology and patient-reported symptoms remains incompletely understood.

Three-dimensional (3D) MRI enables a superior quantitative assessment of cartilage morphology than is possible with conventional two-dimensional (2D) MRI [3]. Previous MRI-based studies have primarily focused on mean cartilage thickness or cartilage volume, which are measures that reflect overall cartilage loss but may overlook spatially localized degeneration [4]. To address this limitation, the cartilage area ratio has been proposed as a complementary metric [5]. Defined as the proportion of a predefined region of interest covered by cartilage, this parameter integrates information about focal cartilage defects and diffuse thinning and is less influenced by body size or joint geometry [6]. Nevertheless, although the cartilage area ratio has demonstrated high reproducibility and sensitivity to region-specific cartilage loss [7], its clinical relevance remains incompletely understood, particularly with respect to patient-reported symptoms.

Patient-reported outcome measures (PROMs) are essential for evaluating symptom severity in knee OA [8]. As one such measure, our research group developed an original Numerical Rating Scale (NRS) questionnaire that provides a comprehensive assessment of knee pain, instability, and mechanical symptoms [9]. Knee OA symptoms are multifactorial and are influenced by cartilage status and by patient factors, such as age and body mass index (BMI) [10]**.** Symptoms can also be affected by related structural elements, such as the medial meniscus, which contributes to load distribution in the medial compartment [11]**.** The purpose of the present study was to investigate the associations between medial femoral and medial tibial cartilage area ratios derived from three-dimensional (3D) MRI analysis and NRS scores in patients undergoing high tibial osteotomy (HTO) for medial knee OA. In this study, the NRS was used as a disease-specific PROM to assess patient-reported symptoms. We also used multivariate analysis to evaluate whether these associations were independent of patient characteristics and radiographic severity, and we examined the robustness of these associations in advanced OA, while further exploring the structural relationship between cartilage area ratio and medial meniscus coverage.

## Materials and methods

### Ethics approval and consent

This study was approved by the Medical Research Ethics Committee of the Institute of Science Tokyo and conducted in accordance with the Declaration of Helsinki.

### Study population

Patients who underwent high tibial osteotomy (HTO) [2] for medial knee OA at our institution were screened for inclusion. Eligible patients were those who had undergone magnetic resonance imaging (MRI) within six months prior to surgery and for whom Numerical Rating Scale (NRS) scores were obtained immediately before surgery. A total of 176 patients met these criteria and were included in the final analysis. The BMI and Kellgren–Lawrence (KL) grade [12] were obtained for each patient and used for subsequent analyses.

### Magnetic resonance imaging

MRI was performed at 3.0 T (Achieva 3.0TX, Philips, Amsterdam, Netherlands) using a dedicated 16-channel transmit/receive knee coil (dStream T/R Knee 16ch coil, Philips). We acquired two-dimensional sagittal fat-suppressed spoiled gradient echo (SPGR) and proton density-weighted (PDW) sequences. The rest time before the MRI scan was approximately 20 min [12–14].

### 3D MRI–based cartilage thickness mapping and ROI segmentation

The MRI analyses were performed using a three-dimensional (3D) image analysis system volume analyzer (SYNAPSE 3D, Collaborative version 6.7; Fujifilm Corporation, Tokyo, Japan) [15–17]. MRI DICOM (Digital Imaging and Communications in Medicine) data were analyzed using software developed by our research group. Approximately 3 min after loading the MRI data, bone and cartilage regions were automatically extracted, and corresponding 3D images were reconstructed. Bone regions and regions of interest (ROIs) were automatically segmented from proton density–weighted (PDW) images, whereas cartilage regions were segmented from spoiled gradient recalled echo (SPGR) images.

**Fig. 1:**
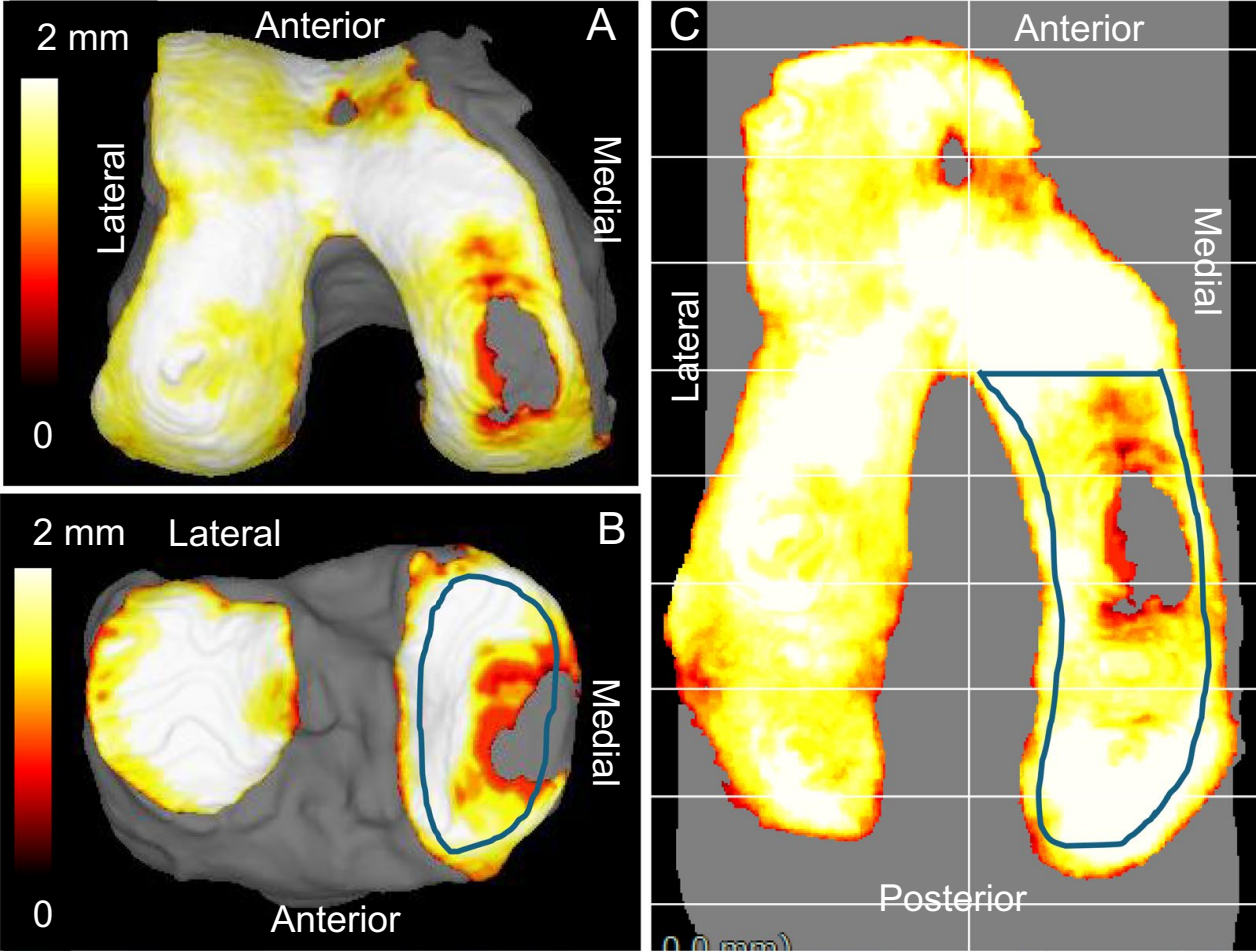
Cartilage thickness mapping and regions of interest for cartilage area ratio. **A** Vertically projected cartilage thickness mapping of the femoral cartilage generated from three-dimensional (3D) MRI analysis. **B** Vertically projected cartilage thickness mapping of the tibial cartilage, with the corresponding medial tibial region of interest (ROI). **C** Radially projected cartilage thickness mapping of the femoral cartilage, with the corresponding medial femoral ROI. Cartilage thickness is displayed using a color scale, with cartilage thickness ≥ 2 mm shown in white. Thinner cartilage is represented by a gradual color change, and areas with complete cartilage loss are shown in gray, indicating exposed subchondral bone. The cartilage area ratio was calculated as the cartilage-covered area divided by the total ROI area

The software provides cartilage thickness mapping by displaying cartilage thickness using a color scale. For vertically projected cartilage analysis, the femoral and tibial cartilages were projected vertically onto a plane along the long axis of each bone (Fig. 1A, B). For radially projected cartilage analysis, the femoral cartilage was projected radially around the intercondylar axis, defined as the line connecting the centers of the medial and lateral femoral condyles. The center of each condyle was determined by approximating the condyle to an ellipse in the lateral view (Fig. 1C). The software automatically generated a closed curve defining the ROI based on the bone contour (Fig. 1B, C) [7]. No manual correction or post-processing was performed after the automated segmentation, and all analyses were conducted using fully automated outputs [17].

### Cartilage area ratio

The cartilage area ratio was calculated within predefined regions of interest (ROIs) in the medial femoral and medial tibial compartments. The ROIs were anatomically defined based on the morphology of the articular bone surface using reconstructed three-dimensional bone models rather than by simply tracing marginal osteophytes. Within each ROI, the proportion of the area covered by segmented cartilage relative to the total ROI area was quantified and used for subsequent analyses [5].

The accuracy of the automatic segmentation performed by this software has been previously validated. Reported Dice similarity coefficients were 0.911 for femoral cartilage and 0.892 for tibial cartilage. For related anatomical structures, Dice similarity coefficients have also been reported as 0.980 for the tibial bone and 0.888 for the medial/lateral tibial plateau ROI [18].

### Meniscus coverage ratio

The medial meniscus (MM), including the anterior and posterior horns, was projected onto a plane perpendicular to the tibial long axis, which served as the reference axis, and the meniscal area was measured on the projected image. The medial cartilage area was automatically defined based on the morphology of the proximal tibia [7]. The MM coverage ratio was calculated as the proportion of the total medial cartilage area covered by the MM, defined as the overlapping area between the MM and the medial cartilage area divided by the total medial cartilage area [19].

### Numerical rating scale (NRS)

Patient-reported symptoms were assessed using the original NRS questionnaire developed by our research group [9]. The questionnaire comprised 10 items (Q1–Q10) designed to assess pain, instability, and mechanical symptoms of the knee (Table 1). Items Q1–Q4 assessed knee pain during daily activities, Q5 assessed knee pain during sports activities (if applicable), Q6–Q8 assessed knee instability during specific activities, and Q9 and Q10 assessed mechanical symptoms, including grinding/noise and catching sensations. Each item was scored on an 11-point scale ranging from 0 (no symptoms) to 10 (worst imaginable symptoms). NRS scores were obtained before surgery. Because responses to Q5 were not available from all participants, this item was excluded from subsequent correlation analyses. A total NRS score was calculated as the sum of Q1–Q4 and Q6–Q10, yielding a maximum possible score of 90, and was used for correlation analyses with cartilage measurements.

**Table 1:** Numerical Rating Scale (NRS) questionnaire items

Item	Description
Q1	Knee pain when walking on flat ground
Q2	Knee pain when sitting still
Q3	Knee pain when starting to move in the morning
Q4	Knee pain when walking up or down stairs
Q5	Knee pain during sports activities (if applicable)
Q6	Instability in the knee when walking on stairs over the past few days
Q7	Instability in the knee when walking on level ground over the past few days
Q8	Instability in the knee when running on level ground over the past few days
Q9	Grinding or noise in the knee when moving it over the past few days
Q10	A sensation of catching when moving the knee over the past few days

### Statistical analysis

All statistical analyses were performed using BellCurve for Excel (Social Survey Research Information Co., Ltd., Tokyo, Japan) and GraphPad Prism 10 (GraphPad Software, Boston, MA, USA). Spearman’s rank correlation coefficients were calculated for the associations among the total NRS score, medial femoral cartilage area ratio, medial tibial cartilage area ratio, body mass index (BMI), and meniscus coverage ratio.

For item-level analyses examining the associations between cartilage area ratio and individual NRS items, nine comparisons (Q1–Q4 and Q6–Q10, with Q5 excluded, as described above) were performed in each figure, and statistical significance was determined using the Holm correction for multiple comparisons with a two-sided α = 0.05. Unadjusted P values are presented in the figures.

Multivariable linear regression analyses were performed with the total NRS score as the dependent variable to determine whether the cartilage area ratio was independently associated with the total NRS score. Separate models were constructed using the medial femoral cartilage area ratio or the medial tibial cartilage area ratio as the main explanatory variable. Age, sex, BMI, and KL grade were included as covariates in each model. Sex was coded as 0 for female patients and 1 for male patients. Standardized β coefficients were calculated to compare the relative contribution of each variable, and variance inflation factors (VIFs) were used to assess multicollinearity.

The robustness of the correlations between the total NRS score and cartilage area ratio was assessed by conducting sensitivity analyses that progressively excluded cases with high cartilage area ratio values. Upper thresholds for the cartilage area ratio were sequentially lowered, and Spearman’s rank correlation analyses were repeated for each threshold.

Scatter plots were generated using Microsoft Excel (Microsoft Office Professional 2021) and GraphPad Prism 10. Except for the item-level analyses with Holm correction described above, a two-sided P value of < 0.05 was considered statistically significant.

## Results

### Patient characteristics

A total of 176 patients who underwent high tibial osteotomy for medial knee OA were included in the analysis (Table 2). The median age was 58 years (range, 19–77 years). Of these patients, 65 were male and 111 were female. The median BMI was 24.6 kg/m^2^ (range, 16.6–43.0 kg/m^2^) in 172 patients. Regarding radiographic severity, the KL grades were 0 in 19 patients, 1 in 35, 2 in 27, 3 in 89, and 4 in 6. BMI data were missing or invalid for four patients.

**Table 2:** Patient characteristics

Characteristic	Value	n
Age, median (range), years	58 (19–77)	176
Sex		176
Male		65
Female		111
BMI, median (range), kg/m^2^	24.6 (16.6–43.0)	172*
KL grade		176
0		19
1		35
2		27
3		89
4		6

^*^Four cases had missing or invalid BMI data

### Cartilage area ratio of the medial femoral and medial tibial regions

The median cartilage area ratio was 0.987 (range, 0.359–1.000) for the medial femur and 0.999 (range, 0.408–1.000) for the medial tibia. The median values were high in both regions. However, substantial inter-individual variability in the cartilage area ratio was observed.

### Correlation between medial femoral cartilage area ratio and NRS score

**Fig. 2:**
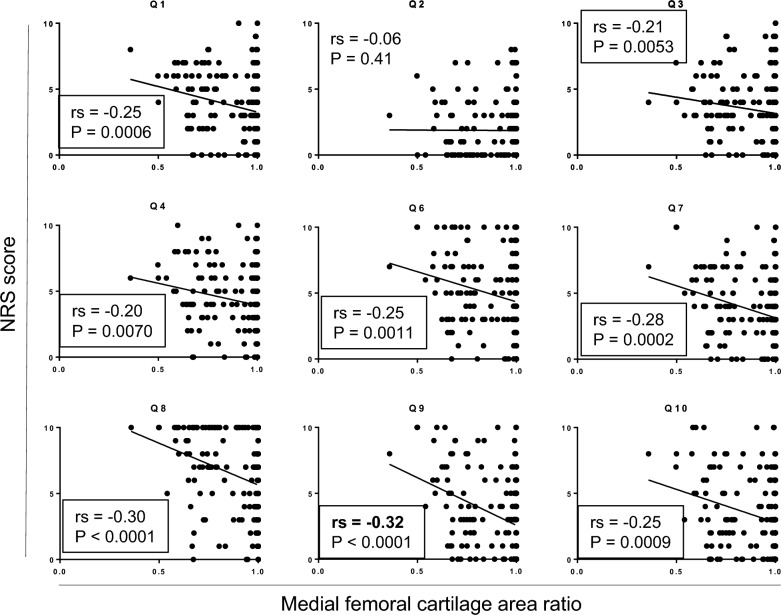
Correlation between medial femoral cartilage area ratio and NRS score. Scatter plots showing the relationship between the medial femoral cartilage area ratio and individual items of the Numerical Rating Scale (NRS) questionnaire (Q1–Q10). Q5, corresponding to knee pain during sports activities (if applicable), was excluded because responses were unavailable for some participants. Each dot represents one patient (n = 176). Solid lines indicate linear trend lines for visualization. Spearman’s rank correlation coefficient (rs) and unadjusted P values are shown in each panel. Statistical significance was determined using the Holm correction for multiple comparisons within this figure (nine comparisons; two-sided α = 0.05). Panels showing significant correlations after Holm correction are highlighted with boxes. The item with the largest absolute correlation coefficient is indicated in bold

Because responses to Q5 were not available from all participants, this item was excluded from subsequent correlation analyses. Significant negative correlations were observed for Q1, Q6, Q8, Q9, and Q10 (Fig. 2). Among these items, those with an absolute correlation coefficient greater than 0.30 were Q8 (instability when running) and Q9 (grinding or noise in the knee).

### Correlation between medial tibial cartilage area ratio and NRS score

**Fig. 3:**
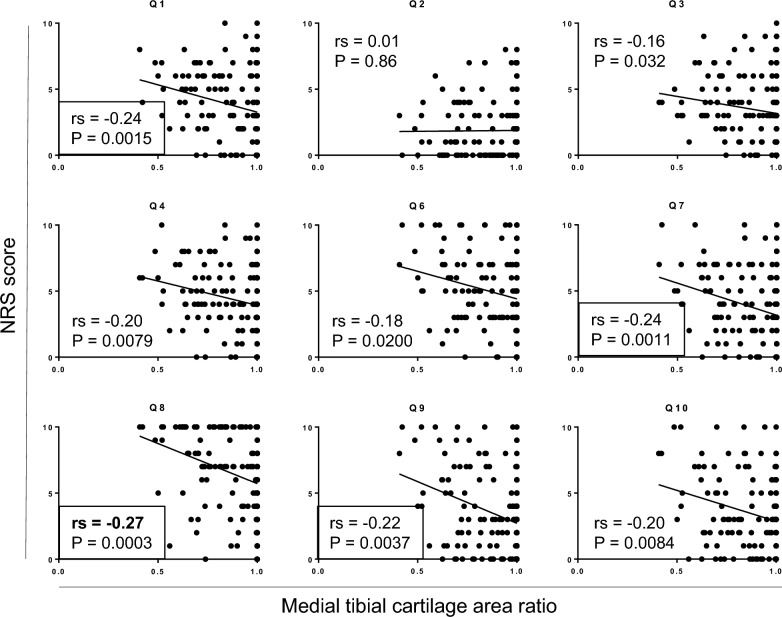
Correlation between medial tibial cartilage area ratio and NRS score. Scatter plots showing the relationship between the medial tibial cartilage area ratio and individual items of the Numerical Rating Scale (NRS) questionnaire (Q1–Q10). Q5, corresponding to knee pain during sports activities (if applicable), was excluded because responses were unavailable for some participants. Each dot represents one patient (n = 176). Solid lines indicate linear trend lines for visualization. The Spearman’s rank correlation coefficient (rs) and unadjusted P values are shown in each panel. Statistical significance was determined using the Holm correction for multiple comparisons within this figure (nine comparisons; two-sided α = 0.05). Panels showing significant correlations after Holm correction are highlighted with boxes. The item with the largest absolute correlation coefficient is indicated in bold

Significant negative correlations between the medial tibial cartilage area ratio and NRS scores were observed for Q1, Q7, and Q8 (Fig. 3). Among these items, the largest absolute correlation coefficient was observed for Q8 (instability when running).

### Correlation between cartilage area ratio and total NRS score

**Fig. 4:**
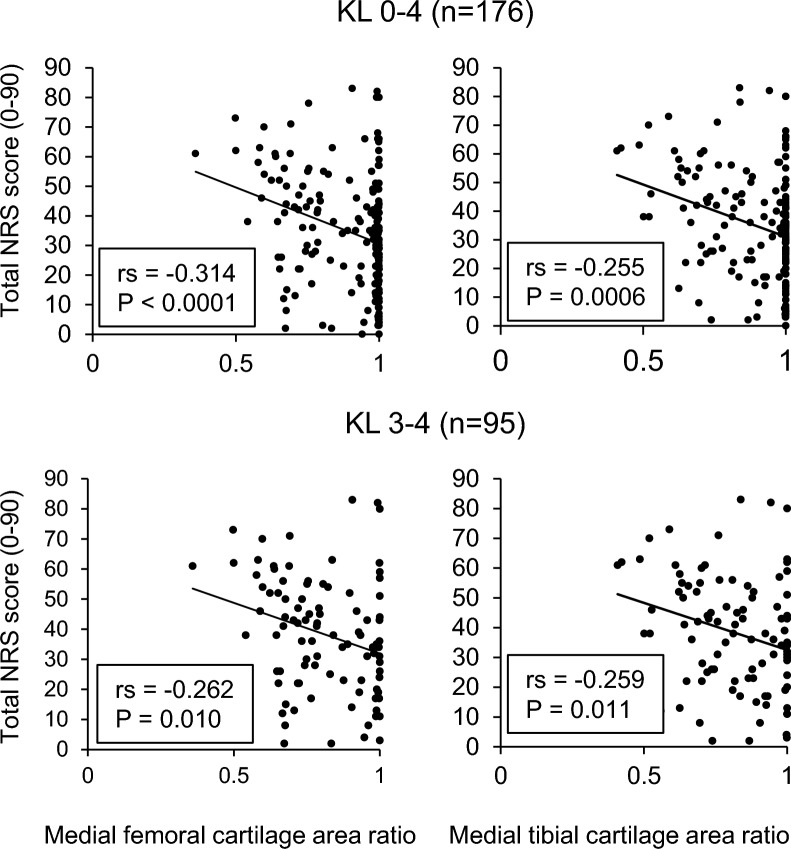
Correlation between cartilage area ratio and total NRS score, including KL-stratified analysis. Scatter plots showing the relationship between cartilage area ratio in the medial femur and medial tibia and the total NRS score, calculated as the sum of Q1–Q4 and Q6–Q10 (range, 0–90). Analyses were performed in the overall cohort (Kellgren–Lawrence [KL] grades 0–4, n = 176) and in the advanced OA subgroup (KL grades 3–4, n = 95). Each dot represents one patient. Solid lines indicate linear trend lines for visualization. The Spearman’s rank correlation coefficient (rs) and P values are shown in each panel

The total NRS score, calculated as the sum of Q1–Q4 and Q6–Q10 (maximum score of 90), showed a significant negative correlation with both the medial femoral and medial tibial cartilage area ratios (Fig. 4). The correlation was stronger for the medial femoral cartilage area ratio (rs =  − 0.31) than for the medial tibial cartilage area ratio (rs =  − 0.26).

### Correlation between cartilage area ratio and total NRS score in advanced OA

In the subgroup of patients with advanced OA (KL grades 3–4, n = 95), the relationship between cartilage area ratio and total NRS score was further examined. Significant negative correlations were observed for both the medial femoral cartilage area ratio (rs =  − 0.26, P = 0.010) and the medial tibial cartilage area ratio (rs =  − 0.26, P = 0.011) (Fig. 4).

### Multivariable analysis and association with BMI

**Fig. 5:**
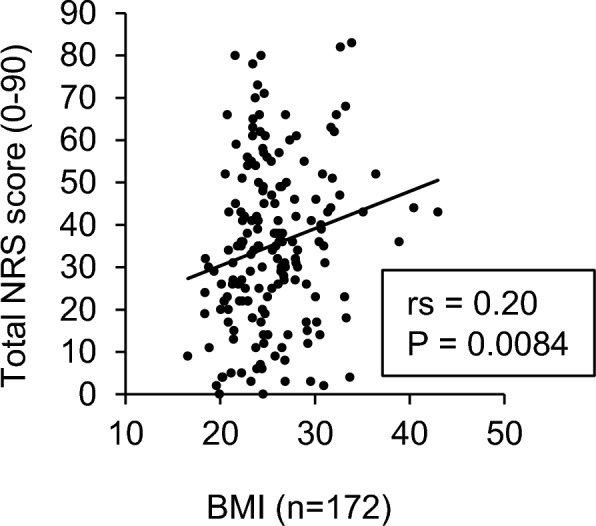
Correlation between body mass index (BMI) and total NRS score. The scatter plot shows the relationship between BMI and the total NRS score, calculated as the sum of Q1–Q4 and Q6–Q10 (range, 0–90). Each dot represents one patient (n = 172). The solid line indicates a linear trend line for visualization. The Spearman’s rank correlation coefficient (rs) and P value are shown

Multivariable linear regression analysis was performed to evaluate whether the cartilage area ratio was associated with the total NRS score after adjustment for age, sex, BMI, and KL grade (Table 3). In the femoral model, the medial femoral cartilage area ratio was significantly associated with the total NRS score (standardized β =  − 0.245, P = 0.007), and BMI was also significantly associated with total NRS score (standardized β = 0.188, P = 0.017). In the tibial model, the medial tibial cartilage area ratio was likewise significantly associated with the total NRS score (standardized β =  − 0.226, P = 0.012), together with the BMI (standardized β = 0.180, P = 0.022). In contrast, age, sex, and KL grade were not significantly associated with the total NRS score in either model. The standardized β coefficients were larger for cartilage area ratio than for BMI in both models. The BMI also showed a weak but significant positive correlation with the total NRS score (rs = 0.20, P = 0.0084; Fig. 5). Variance inflation factor values ranged from 1.05 to 1.32.

**Table 3:** Multivariable linear regression analysis for total NRS score

Variable	β (95% CI)	P value	Standardized β	VIF
*Femoral cartilage area ratio model*				
Medial femoral cartilage area ratio	-31.87 (-54.95 to -8.80)	0.007	-0.245	1.32
Age	0.05 (-0.21 to 0.32)	0.690	0.033	1.18
Sex				
(male = 1, female = 0)	-3.00 (-8.97 to 2.97)	0.323	-0.073	1.05
BMI	0.83 (0.15 to 1.50)	0.017	0.188	1.14
KL grade	1.35 (-1.76 to 4.46)	0.392	0.076	1.21
Adjusted R^2^	0.107			
*Tibial cartilage area ratio model*				
Medial tibial cartilage area ratio	-29.04 (-51.73 to -6.35)	0.012	-0.226	1.29
Age	0.04 (-0.23 to 0.31)	0.751	0.027	1.17
Sex				
(male = 1, female = 0)	-2.74 (-8.71 to 3.23)	0.366	-0.066	1.05
BMI	0.79 (0.11 to 1.47)	0.022	0.180	1.13
KL grade	1.60 (-1.49 to 4.69)	0.308	0.091	1.20
Adjusted R^2^	0.102			

Multivariable linear regression analyses were performed with total NRS score as the dependent variable. In the femoral model, the independent variables included medial femoral cartilage area ratio, age, sex, body mass index (BMI), and Kellgren–Lawrence (KL) grade. In the tibial model, the medial tibial cartilage area ratio was entered instead of medial femoral cartilage area ratio. Sex was coded as male = 1 and female = 0. Cases with invalid BMI values were excluded, resulting in 172 patients in the final analysis. Standardized β coefficients are presented to facilitate comparison of effect sizes across variables. The variance inflation factor (VIF) was calculated to assess multicollinearity, and no substantial multicollinearity was observed

### Relationships of meniscus coverage ratio with cartilage area ratio and total NRS score

**Fig. 6:**
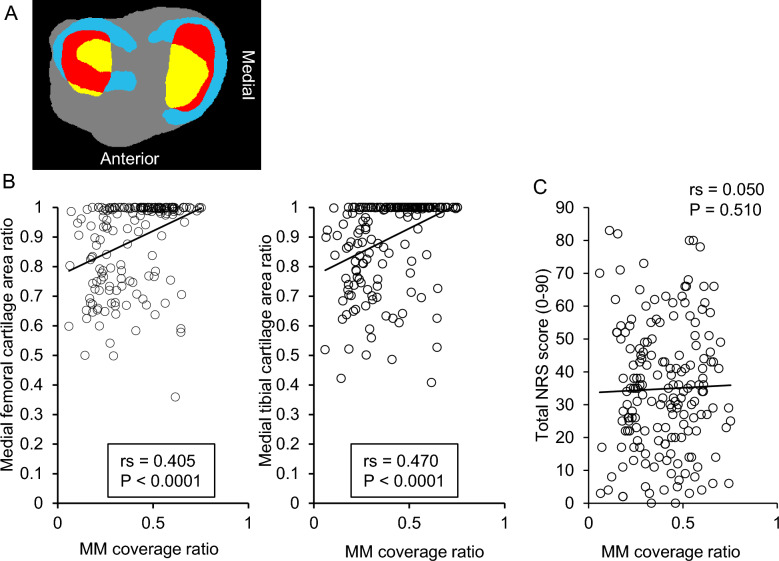
Relationships of meniscus coverage ratio with cartilage area ratio and total NRS score. **A** Schematic illustration of the meniscus coverage ratio. On the tibial articular surface, the medial and lateral menisci are shown for anatomical orientation. The medial tibial cartilage area is shown in red and yellow, the medial meniscus (MM) in red and blue, and their overlapping area in red. Although both menisci are depicted, the meniscus coverage ratio was calculated using the MM only, as the overlapping area between the MM and the medial tibial cartilage area divided by the total medial tibial cartilage area. **B** Scatter plots showing the relationship between meniscus coverage ratio and cartilage area ratio in the medial femur (left) and medial tibia (right). Each dot represents one patient. Solid lines indicate linear trend lines for visualization. Spearman’s rank correlation coefficient (rs) and P values are shown in each panel. **C** Scatter plot showing the relationship between meniscus coverage ratio and total NRS score. Each dot represents one patient. The solid line indicates a linear trend line for visualization. The Spearman’s rank correlation coefficient (rs) and P value are shown

The relationship between meniscus coverage ratio and cartilage area ratio was evaluated. The meniscus coverage ratio was defined as the proportion of the medial tibial cartilage area covered by the medial meniscus (Fig. 6A). The meniscus coverage ratio showed significant positive correlations with the cartilage area ratio in both the medial femur (rs = 0.41, *P* < 0.0001) and medial tibia (rs = 0.47, *P* < 0.0001) (Fig. 6B). In contrast, the meniscus coverage ratio was not significantly correlated with the total NRS score (rs = 0.050, *P* = 0.510) (Fig. 6C).

### Sensitivity analyses for ceiling effects

The cohort included 89 patients with KL grade 3, and the median cartilage area ratio was 0.987 in the medial femur and 0.999 in the medial tibia. Sensitivity analyses were therefore performed using progressively lower upper thresholds for the cartilage area ratio (Table 4). For the medial femoral cartilage area ratio, the negative correlation with the total NRS score remained statistically significant down to a threshold of 0.700. For the medial tibial cartilage area ratio, statistical significance was maintained down to 0.800 but was not retained at 0.700.

**Table 4:** Sensitivity analyses of the correlations between total NRS score and cartilage area ratio using progressively lower upper thresholds

Threshold	Medial femoral cartilage area ratio			Medial tibial cartilage area ratio		
	n	rs*	P value	n	rs*	P value
≤ 1.000	176	-0.314	< 0.001	176	-0.255	< 0.001
≤ 0.999	135	-0.194	0.024	90	-0.336	0.001
≤ 0.990	98	-0.343	< 0.001	80	-0.262	0.019
≤ 0.900	62	-0.293	0.021	62	-0.299	0.018
≤ 0.800	51	-0.317	0.024	41	-0.318	0.043
≤ 0.700	27	-0.421	0.029	23	-0.403	0.056
≤ 0.600	9	-0.217	0.576	9	-0.084	0.831

Sensitivity analyses were performed using progressively lower upper thresholds for the medial femoral and medial tibial cartilage area ratios to assess the potential influence of ceiling effects on the correlations with total NRS

^*^Spearman’s rank correlation coefficient

## Discussion

The cartilage area ratio derived from three-dimensional (3D) MRI was significantly associated with patient-reported symptoms in patients undergoing high tibial osteotomy (HTO) for medial knee OA. The associations were observed in both the medial femoral and medial tibial compartments, with slightly stronger correlations in the medial femur. Notably, cartilage area ratio showed closer relationships with instability-related and mechanical symptoms than with pain-related symptoms, while the association with the total Numerical Rating Scale (NRS) score remained significant in both compartments. These findings suggest that the cartilage area ratio reflects structural joint abnormalities that are linked to overall symptom burden, but particularly to functional impairment related to joint instability and mechanical dysfunction.

The closer association of the cartilage area ratio with instability and mechanical symptoms than with pain is clinically plausible. Pain in knee OA is multifactorial and may originate from the synovium, subchondral bone, periarticular soft tissues, and central pain processing [10], which may weaken its direct relationship with cartilage morphology. In contrast, instability and mechanical symptoms, such as grinding or noise, are more directly influenced by alterations in joint congruity and load distribution [20], which depend on the extent of cartilage coverage in the medial compartment. Therefore, by quantifying the proportion of cartilage-covered surface, the structural changes relevant to these symptoms may be better captured by the cartilage area ratio than by conventional global measures, such as the mean cartilage thickness or cartilage volume [21].

Instability during running showed the strongest association with the cartilage area ratio in both the medial femoral and medial tibial compartments. The joint contact stresses and dynamic loading are higher when imposed by running than by level walking [22], potentially amplifying the functional consequences of localized cartilage loss. Reduced cartilage coverage may compromise joint surface conformity and shock absorption, leading to perceived instability during high-demand activities. Clinically, a preoperative assessment of the cartilage area ratio may help surgeons identify patients with higher functional demands while also explaining the closer relationship of symptom improvement after HTO to dynamic symptoms, such as instability during activity, than to pain at rest.

The slightly stronger associations observed in the medial femoral compartment than in the medial tibial compartment may reflect the greater biomechanical contribution of the femoral condyle to dynamic knee joint function. Because of the femoral compartment’s curvature and its role in articulation during flexion and rotation, focal cartilage defects may have greater effects on contact stress distribution and joint kinematics during weight-bearing activities [20, 23]. In addition, loading of the femoral cartilage may be more strongly affected by meniscal extrusion and malalignment, both of which alter the load distribution across the medial compartment [24]. These biomechanical factors may help explain why the femoral cartilage area ratio showed slightly stronger associations with clinical symptoms, particularly those related to stability and mechanical function.

The subgroup analysis of advanced OA further demonstrated that the association between cartilage area ratio and total NRS score was preserved in patients with Kellgren–Lawrence grades 3–4. This suggests that the cartilage area ratio retains clinical relevance, even in advanced OA, despite the greater structural heterogeneity typically observed at this stage. In such settings, conventional global measures may be less sensitive to residual variations in cartilage morphology [25]. By quantifying the extent of cartilage coverage within the medial compartment, the cartilage area ratio may capture meaningful structural differences related to symptom burden, even among patients with advanced OA.

Multivariable analysis further supported the clinical relevance of the cartilage area ratio. The persistence of the association after adjustment for age, sex, BMI, and KL grade indicates that the cartilage area ratio captures information related to symptom severity beyond these conventional clinical and radiographic factors. In both the femoral and tibial models, the cartilage area ratio remained significantly associated with the total NRS score. The BMI was also significantly associated with total NRS score, indicating that body weight contributes to symptom burden [26]. Notably, the larger standardized β coefficients for cartilage area ratio than for BMI in both models suggest that the association with symptom severity may be greater for the structural cartilage status than for body mass in this cohort. These findings indicate that the cartilage area ratio provides clinically relevant information beyond that achievable based on conventional patient characteristics and radiographic severity.

Analysis of the meniscus coverage ratio provided additional insight into the structural relevance of the cartilage area ratio. The meniscus coverage ratio showed moderate positive correlations with the cartilage area ratio in both the medial femoral and medial tibial compartments, indicating a close structural relationship between meniscal coverage and cartilage morphology in the medial compartment. In contrast, the meniscus coverage ratio was not significantly associated with the total NRS score. Because the meniscus coverage ratio was associated with the cartilage area ratio [19] but not with the total NRS score, the meniscal morphology may contribute to symptoms indirectly through its relationship with cartilage structural changes rather than as a direct determinant of symptom severity. Thus, the association between the cartilage area ratio and symptoms is unlikely to be explained solely by meniscal morphology.

The relatively high median cartilage area ratio values, despite the predominance of KL grade 3 knees, suggest that radiographic OA severity does not necessarily indicate extensive loss of cartilage coverage, particularly in HTO candidates. This distribution supported the need for sensitivity analyses using progressively lower upper thresholds to address potential ceiling effects. The sensitivity analyses demonstrated that the inverse correlations between cartilage area ratio and total NRS score persisted when cases with high cartilage area ratio values were progressively excluded. This supports the interpretation that the observed associations were not merely an artifact of ceiling effects caused by clustering near the upper limit of the cartilage area ratio. In the medial femur, the association remained significant even when the upper threshold was lowered to 0.700. In contrast, significance in the medial tibia was maintained only to a threshold of 0.800. The loss of statistical significance at the lowest thresholds, particularly in the medial tibia, is more likely to reflect the reduced sample size and loss of statistical power than a true absence of association [27].

Several limitations should be acknowledged. First, the observed correlations were modest, with absolute correlation coefficients reaching only approximately 0.32, even at the item level, indicating that the cartilage area ratio explained only a limited proportion of the variability in patient-reported symptoms. This is not unexpected, as symptoms in knee osteoarthritis are multifactorial and influenced by factors beyond cartilage morphology, including synovial inflammation, subchondral bone abnormalities, lower limb alignment, muscle strength, and other periarticular structural aspects [10, 28]. Second, although we added analyses of the meniscus coverage ratio, other potentially relevant structural factors, particularly bone marrow lesions, were not evaluated [29]. Third, the study population was limited to patients undergoing HTO for medial knee osteoarthritis at a single institution, so the cases represented a relatively specific phenotype characterized by varus alignment and predominant medial compartment disease. Therefore, these findings may not be directly generalizable to broader osteoarthritis populations, other surgical indications, or different healthcare settings. Fourth, because of the cross-sectional design, causal relationships between the reduced cartilage area ratio and symptom severity cannot be determined [30]. Finally, Q5 was excluded because responses were unavailable for some patients, but this exclusion may have reduced sensitivity for detecting associations with sports-related pain.

Despite these limitations, the present findings suggest that the cartilage area ratio is a complementary structural imaging marker that is modestly, but consistently, associated with patient-reported symptoms in medial knee OA, while accounting for only a limited proportion of overall symptom variability. Its preferential association with instability and mechanical symptoms suggests that it reflects functional joint impairment linked to structural degeneration rather than nonspecific symptom burdens. Furthermore, because patients undergoing HTO are typically relatively high-functioning and physically active despite medial compartment degeneration, dynamic symptoms during higher-demand activities may be more clinically prominent than resting pain. This cohort characteristic may partly explain why the cartilage area ratio showed stronger associations with instability-related symptoms than with pain at rest. Clinically, this may be relevant when evaluating relatively high-functioning patients who are candidates for HTO because a lower cartilage area ratio may indicate a greater likelihood of load-dependent or instability-related symptoms associated with structural cartilage loss.

## Conclusion

Lower cartilage area ratios in the medial compartment were associated with greater patient-reported symptom severity in medial knee OA, particularly regarding instability during running and mechanical symptoms. These findings indicate that the cartilage area ratio captures structural abnormalities related to functional joint impairment beyond pain alone. Although the observed associations were modest, the cartilage area ratio may serve as a useful complementary structural imaging biomarker for assessing disease severity in medial knee OA.

## Data Availability

The datasets used and/or analyzed during the current study are available from the corresponding author on reasonable request.

## Abbreviations

OA: Osteoarthritis
MRI: Magnetic resonance imaging
3D: Three-dimensional
2D: Two-dimensional
PROMs: Patient-reported outcome measures
HTO: High Tibial Osteotomy
NRS: Numerical Rating Scale
SPGR: Spoiled gradient recalled echo
PDW: Proton Density–Weighted
ROI: Region of interest
MM: Medial meniscus
rs: Spearman’s rank correlation coefficient
DICOM: Digital imaging and communications in medicine
